# Rethinking white matter–tumor interaction: a tractography based analysis of associations between fractional anisotropy and morphometry in the IFOF and arcuate fasciculus

**DOI:** 10.1007/s11060-026-05691-4

**Published:** 2026-07-07

**Authors:** Roberto Altieri, Lorenzo Ugga, Andrea Bianconi, Stefano Caneva, Ferdinando Caranci, Giovanni Cirillo, Fabio Cofano, Sergio Corvino, Oreste de Divitiis, Giuseppe Maria Della Pepa, Donatella Franco, Ciro De Luca, Pietro Fiaschi, Gianluca Galieri, Diego Garbossa, Giuseppe La Rocca, Salvatore Marino, Edoardo Mazzucchi, Grazia Menna, Antonio Mezzogiorno, Alberto Morello, Alessandro Olivi, Michele Papa, Daniela Pacella, Rosellina Russo, Giovanni Sabatino, Giovanna Sepe, Assunta Virtuoso, Giovanni Vitale, Giuseppe Vitale, Gianluigi Zona, Manlio Barbarisi

**Affiliations:** 1 Multidisciplinary Department of Medical-Surgical and Dental Specialties, University of Campania Luigi Vanvitelli, 80131 Naples, Italy; 2 Department of Advanced Medical and Surgical Sciences, School of Medical Sciences, University of Campania Luigi Vanvitelli, P.zza L. Miraglia 2, 80138 Naples, Italy; 3 Department of Neuroscience, Rehabilitation, Ophthalmology, Genetics, Maternal and Child Health (DINOGMI), University of Genova, 16132 Genova, Italy; 4 Department of Neurosurgery, IRCCS Ospedale Policlinico San Martino, 16132 Genova, Italy; 5 Laboratory of Morphology of Neuronal Network, Department of Public Medicine, University of Campania Luigi Vanvitelli, 80138 Naples, Italy; 6 Neurosurgery Unit, Department of Neuroscience Rita Levi Montalcini, University of Turin, 10126 Turin, Italy; 7 Division of Neurosurgery, Ospedale del Mare Hospital, 80147 Naples, Italy; 8 Department of Neuroscience and Reproductive and Odontostomatological Sciences, Neurosurgical Clinic, University of Naples Federico II, Via Pansini 5, 80131 Naples, Italy; 9 Institute of Neurosurgery, Fondazione Policlinico Universitario A. Gemelli IRCCS, Catholic University, 00168 Rome, Italy; 10 Neurosurgical Training Center and Brain Research, Mater Olbia Hospital, 07026 Olbia, Italy; 11 Department of Neurosurgery, IRCCS Regina Elena National Cancer Institute, 00144 Rome, Italy; 12 Department of Public Health, University of Naples Federico II, Naples, Italy; 13 Department of Radiology, Neuroradiology Unit, Fondazione Policlinico Universitario A. Gemelli IRCCS, 00168 Rome, Italy; 14 Neurosurgery Unit, Regional Hospital San Carlo, Potenza, Italy; 15 Radiology Department, CTO Hospital, AORN dei Colli, 80131 Naples, Italy

**Keywords:** Glioblastoma, Low grade glioma, Metastasis, Meningioma, Arcuate fascicle, Connectome

## Abstract

**Background:**

White matter (WM) tract involvement represents a critical determinant of functional outcome in neuro-oncology. Although substantial advances in diffusion tensor imaging (DTI) and intraoperative mapping have improved characterization of WM organization, the relationship between microstructural integrity and morphometric alterations of associative fasciculi in different tumor histotypes remains incompletely understood. We investigated the association between fractional anisotropy (FA), volumetric and length parameters of the inferior fronto-occipital fasciculus (IFOF) and arcuate fasciculus (AF) across distinct intracranial tumor types.

**Methods:**

In this multicenter retrospective study, 156 patients undergoing surgery for brain tumors were included. All lesions were located in proximity to the IFOF or AF and underwent preoperative deterministic tractography based on standardized DTI protocols. Linear mixed-effects models, adjusted for relevant covariates, assessed the association between FA and tract morphometry, including interaction terms for tumor type and hemisphere.

**Results:**

Higher FA was strongly associated with both AF and IFOF tract volume (adjusted *p* < 0.001), whereas associations with tract length were weaker and not consistently significant. The association between FA and IFOF volume appeared stronger in gliomas than in metastases or meningiomas, suggesting potential histotype-related difference in WM involvement. The healthy hemisphere showed higher FA and morphometric measures than the tumor-affected side.

**Conclusions:**

FA was significantly associated with morphometric features of WM tract, particularly in infiltrative tumors. Integrating FA into preoperative assessment may enhance surgical planning and support strategies aimed at preserving functional connectivity.

## Introduction

Over the past few decades, there has been a growing interest among neurosurgeons in the study of white matter anatomy [1–5]. Despite the remarkable advances achieved through both non-invasive (tractography) and invasive (intraoperative brain mapping) techniques, several aspects of the organization and interactions among white matter tracts remain to be fully elucidated. In particular, the relationship between different fiber bundles and the heterogeneous growth patterns of various brain tumor histotypes continues to be a subject of investigation.

Historically, it was hypothesized that white matter represented a preferential route for glioma dissemination and infiltration. However, recent evidence has challenged this view, demonstrating that glioma progression does not strictly follow white matter pathways [6–8]. Conversely, brain metastases, which were long considered to be foreign bodies within the cerebral parenchyma, have been shown to establish complex interactions with the surrounding microenvironment. Notably, the discovery of functional GABAergic synapses between metastatic and neuronal cells has revealed a novel mechanism through which neuronal activity can promote metastatic growth [9, 10].

In our previous studies, we described morphometric alterations of the inferior fronto-occipital fasciculus (IFOF) and the arcuate fasciculus (AF) in relation to different tumor histotypes [11, 12]. Given that fractional anisotropy (FA) is a recognized index of white matter integrity, we aimed in the present work to investigate whether FA directly influences other morphometric and neuroimaging characteristics of the IFOF and AF.

## Materials and methods

### Study design and patient population

A retrospective multicenter study was conducted, in accordance with the Declaration of Helsinki, across six Italian Neurosurgical Units: two in Northern Italy (University of Turin; I.R.C.C.S. Ospedale Policlinico San Martino, Genoa), two in Central Italy (Università Cattolica del Sacro Cuore, Rome; Mater Olbia Hospital, Olbia), and two in Southern Italy (University of Campania “Luigi Vanvitelli”, Naples; A.O.R. San Carlo, Potenza).

This study was based exclusively on retrospectively collected, fully anonymized clinical and imaging data derived from routine diagnostic and surgical procedures performed as part of standard clinical care. According to institutional policies and applicable regulations, formal Ethics Committee approval was not required for this retrospective analysis. All patients provided written informed consent for the diagnostic and therapeutic procedures performed and for the potential use of their anonymized clinical data for research purposes.

Medical records of 1,294 patients who underwent surgery for intracranial glioblastoma (GBM), low-grade glioma (LGG), brain metastases, or meningioma between January and December 2023 were retrospectively reviewed.

**Fig. 1:**
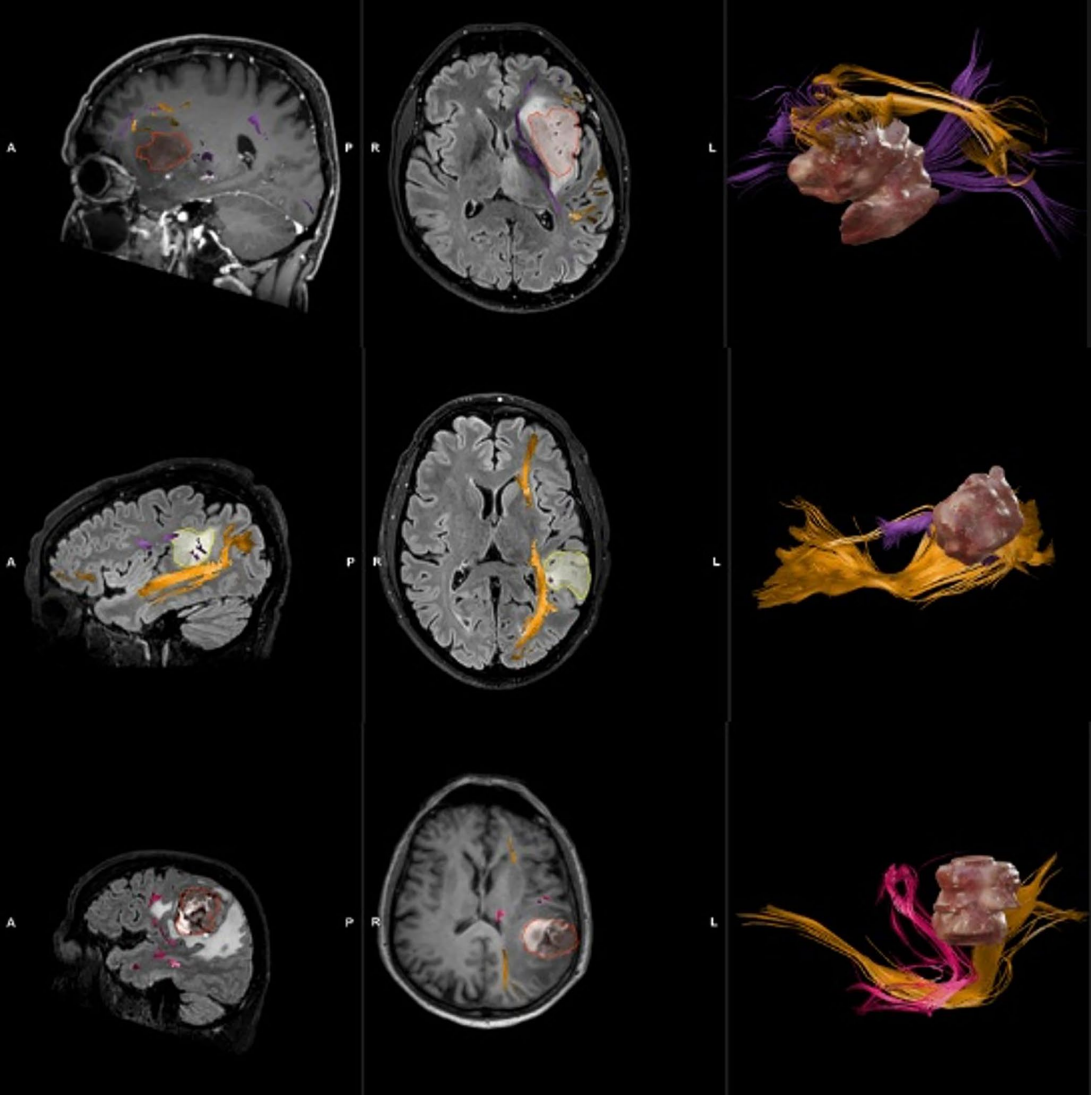
GBM (upper row), LGG (middle row), brain metastasis (lower row)

Inclusion criteria were: (i) age ≥ 18 years; (ii) histological and/or molecular diagnosis confirming GBM, LGG, brain metastasis, or WHO grade I meningioma; (iii) availability of complete preoperative neuroimaging, including contrast-enhanced magnetic resonance imaging (MRI) with T1-weighted, FLAIR, and Diffusion Tensor Imaging (DTI) sequences; and (iv) tumor location in proximity to the IFOF or AF. Tumors were considered to be in proximity to the IFOF or AF when preoperative DTI tractography demonstrated direct contact with, displacement of, distortion of, or infiltration into the course of either tract (Fig. 1).

### MRI acquisition and DTI analysis

MRI scans were performed using 1.5 Tesla systems (A.O.R. San Carlo) and 3 Tesla systems (academic institutions).

DTI data were acquired using 32 diffusion directions at the University of Turin, A.O.R. San Carlo, University of Campania “Luigi Vanvitelli,” and Mater Olbia Hospital; 60 directions at I.R.C.C.S. Ospedale Policlinico San Martino; and 64 directions at Università Cattolica del Sacro Cuore.

Given the retrospective multicenter design, complete acquisition parameters (including b-values, voxel dimensions, TR/TE, number of b0 images, and preprocessing details) were not consistently available across all participating institutions. Tractography analyses were therefore performed using the diffusion datasets available in routine clinical practice at each center and reconstructed using a standardized post-processing workflow within the Brainlab Elements platform.

Preoperative tractography reconstructions were assessed by a multidisciplinary team of neurosurgeons, neuroradiologists, and neuroanatomists using Elements Fibertracking and SmartBrush software (Brainlab AG, Munich, Germany). The IFOF and AF were reconstructed bilaterally for each subject.

Regions of interest (ROIs) were manually placed following the anatomical landmarks described by Feconja et al.—within the frontal lobe (subcallosal and ventral premotor cortices) and the occipital lobe (lateral occipital cortex). Deterministic tractography was used to generate streamlines connecting these regions [13, 14].

Tractography parameters included a minimum streamline length of 50 mm and a FA threshold of 0.15. Streamlines traversing the extreme capsule were retained, whereas those deviating toward adjacent fiber systems were excluded. To minimize interindividual variability, all reconstructions were normalized to a standard anatomical template.

For each fascicle, both in the tumor-affected and contralateral hemispheres, the following parameters were measured: tract volume, mean FA, and tract length. FA threshold was set to 0.15, and the maximum turning angle to 60°, in accordance with default recommendations of the Elements software. Tract volume was defined as the total volume occupied by the reconstructed tract generated by the Brainlab Elements Fibertracking software and was expressed in cm³. Tract length was defined as the mean streamline length of all reconstructed streamlines belonging to the tract and was expressed in millimeters (mm). Both measurements were automatically calculated by the software following tract reconstruction.

Because deterministic tractography relies on predefined FA thresholds for streamline propagation, FA values may influence tract reconstruction itself. In particular, reduced FA may contribute to premature streamline termination and consequently to lower reconstructed tract volumes. Therefore, the relationship between FA and tract morphometric measures should be interpreted as reflecting both underlying biological changes in white matter integrity and methodological characteristics inherent to diffusion tractography reconstruction.

### Statistical analysis

Data are reported as mean (SD) or as frequencies (percentage). Crude and adjusted linear mixed-effects regression models were used to analyze the association between AF/IFOF FA and the outcomes AF/IFOF length or volume accounting also for the potential confounding effect of the other covariates of interest (i.e. age, sex, histotype, side). For all regressions, only variables that were significantly associated with the outcome at crude models were added to the adjusted model. Patient’s ID was considered as a random effect (random intercept only). To investigate the moderator effect of the tumor type and of the healthy/affected side measurement on the relationship between FA and each outcome, two-way interaction terms were added separately to distinct mixed-effects models when these covariates were significant at the adjusted models. Normality of the residuals for all models was checked by inspecting each model’s Q-Q plots. Results are reported as unstandardized beta coefficients with 95% confidence intervals (C.I.). In order to investigate whether differences in FA between healthy and affected sides were correlated with respective differences for other variables of interest, delta measurements were obtained subtracting affected side measurements from healthy side measurements and Pearson correlation was used to quantify the strength of the relationships. All analyses were conducted using R statistical software version 4.4.0. A two-tailed p-value < 0.05 was considered significant.

## Results

During the study period, a total of 1,294 patients underwent neuro-oncological surgery across the participating institutions. Of these, 156 patients met the inclusion criteria. Among the selected cohort, 98 patients (63%) were diagnosed with glioblastoma (GBM), 26 (17%) with low-grade glioma (LGG), 18 (12%) with brain metastasis, and 14 (9%) with meningioma.

The study population consisted of 83 males (53%) and 73 females (47%), with a mean age of 59 years. Tumor lateralization analysis revealed that 109 patients (70%) had lesions located in the left cerebral hemisphere, while 47 (30%) presented with right-sided lesions. Consistent with the inclusion criteria, all tumors demonstrated direct anatomical relationships with the IFOF or AF on preoperative tractography.

Regarding tumor volumetrics, the mean volume of the enhancing nodule (EN) was 25 cm³, while the mean FLAIR hyperintense volume beyond the EN measured 49 cm³.

Morphometric analysis of the AF demonstrated an average tract length of 102 mm on the healthy hemisphere and 100 mm on the tumor-affected side. Mean FA values were 0.46 and 0.44, respectively. The mean tract volume was 22 cm³ in the healthy hemisphere and 16 cm³ in the tumor-affected hemisphere. The IFOF mean length of the of healthy hemisphere and tumor-affected hemisphere was respectively 118 mm and 112 mm. The mean FA of healthy side and tumor-affected side was 0.51 and 0.42, respectively. The mean IFOF volume of healthy side and tumor-affected side was 39 cm3 and 28 cm3 respectively.

Crude and adjusted regressions to investigate the determinants of AF volume reported in Table 1 revealed that AF mean FA and healthy side are directly associated with the outcome (respectively AF mean FA adj. β = 54, *p* < 0.001 and healthy vs. affected side adj. β = 4.3, *p* < 0.001), while Meningioma was associated with a lower AF volume (Meningioma vs. GBM adj. adj. β = -7.1, *p* = 0.001). Interactions between AF, mean FA and side group and between AF mean FA and tumor type tested with separate models were not significant.

Concerning the determinants AF mean length (Table 2), AF and IFOF mean FA were not associated with the outcome at univariable regressions, and only side group and Meningioma were significantly related (respectively healthy vs. affected side adj. β = 2.3, *p* = 0.032 and Meningioma vs. GBM adj. β = 22, *p* < 0.001).

**Fig. 2:**
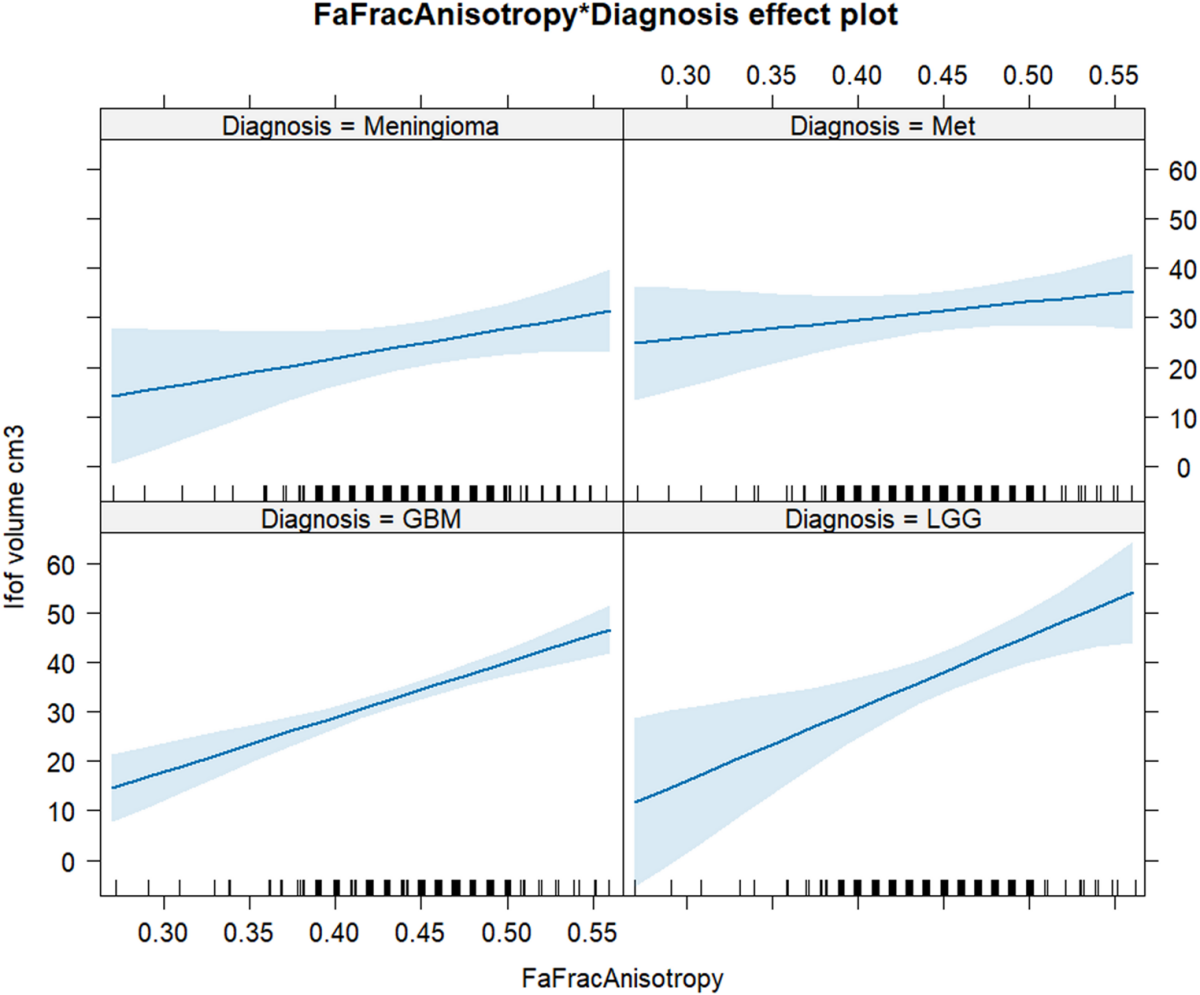
Plot displaying the marginal effects of the interaction between AF mean FA and tumor type on the outcome IFOF volume (cm3) at multivariable mixed-effects linear regression adjusted by group and age with patient’s ID as random effect

For the outcome IFOF volume, both crude and adjusted regressions revealed that AF mean FA and healthy side are positively associated with the outcome (respectively AF mean FA adj. β = 90, *p* < 0.001 and healthy vs. affected side adj. β = 8.4, *p* < 0.001), while Meningioma was associated with a lower IFOF volume (Meningioma vs. GBM adj. adj. β = -9.6, *p* = 0.001) (Table 3). We also investigated with separate models the interactions between AF mean FA and side group and interaction between AF mean FA and tumor type. The latter was significant. As shown in Fig. 2, in LGG and GBM tumors, the direct relationship between AF mean FA and IFOF volume is steeper and thus more pronounced than in Met and Meningioma diagnoses, where the relationship is much less marked. The coefficients are adjusted by age and group with patient’s ID as random effect.

Similar results to AF mean length were found investigating the determinants of IFOF mean length (Table 4). In fact AF mean FA was only associated with the outcome at univariable regression, but such association was not confirmed by the adjusted model, where exclusively side group and Meningioma were significantly associated (respectively healthy vs. affected side adj. β = 5.2, *p* < 0.001 and Meningioma vs. GBM adj. β = 41, *p* < 0.001).

**Fig. 3:**
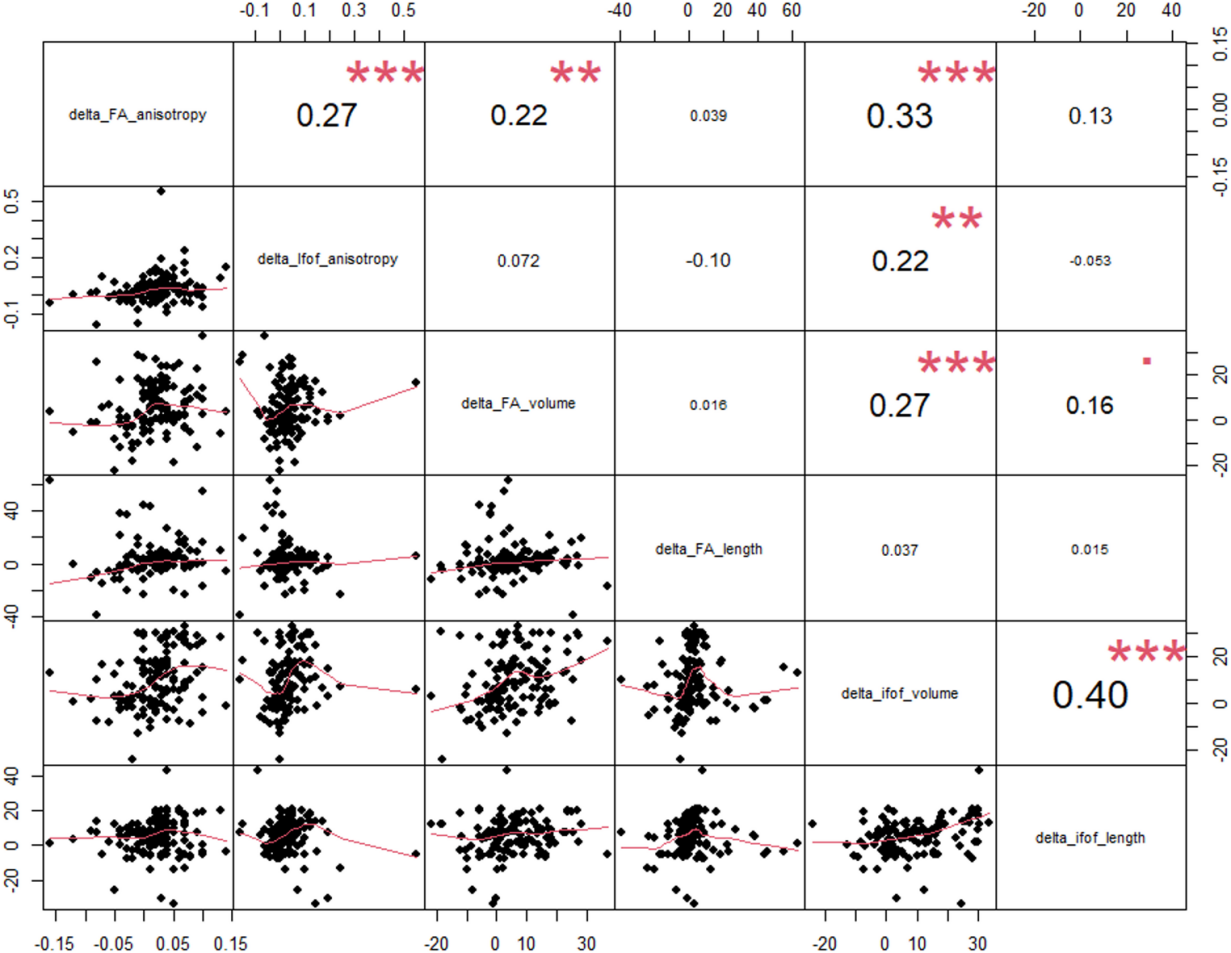
Correlation plot displaying the relationships between the variables of interest calculated as paired differences between healthy and affected sides (delta)

We defined delta measurements as the difference between healthy side measurements and affected side measurements. Correlations between all pairs of variables for volumes, length and FA deltas are displayed in Fig. 3. As can be evidenced, scatterplot and coefficients reveal only weak or modest correlations with FA variables. However it is interesting to highlight the significant correlations between both delta AF mean FA and delta IFOF mean FA with delta IFOF volume (respectively *r* = 0.33, *p* < 0.01 and *r* = 0.22, *p* < 0.01) and also between delta AF mean FA and delta FA volume (*r* = 0.22, *p* < 0.01) (Fig. 3).

**Table 1:** Univariable and multivariable mixed-effects linear regressions investigating the variables associated with AF volume (cm3). (*) AF mean FA betas are reported per 0.1 increase

Outcome: AF volume (cm3)	Univariable			Multivariable		
Characteristic	Beta	95% CI	*p*-value	Adj. Beta	95% CI	*p*-value
IFOF mean FA	1.3	-0.39, 3.0	0.131			
AF mean FA*	7.3	4.9, 9.8	**< 0.001**	5.4	2.9, 7.9	**< 0.001**
Group						
Affected	—	—		—	—	
Healthy	5.4	3.9, 7.0	**< 0.001**	4.3	2.6, 5.9	**< 0.001**
Sex						
Female	—	—				
Male	2.4	-0.10, 4.9	0.060			
Age	-0.06	-0.16, 0.04	0.222			
Diagnosis						
GBM	—	—		—	—	
LGG	3.1	-0.20, 6.4	0.065	2.4	-0.84, 5.6	0.147
Meningioma	-6.3	-11, -2.0	**0.004**	-7.1	-11, -2.9	**0.001**
Met	-2.4	-6.3, 1.4	0.213	-2.9	-6.6, 0.86	0.131
Side						
Left	—	—				
Right	-0.60	-3.3, 2.1	0.662			

Significant *p*-values are highlighted in bold

**Table 2:** Univariable and multivariable mixed-effects linear regressions investigating the variables associated with AF mean length (mm). (*) AF mean FA betas are reported per 0.1 increase

Outcome: AF mean length (mm)	Univariable			Multivariable		
Characteristic	Beta	95% CI	*p*-value	Adj. Beta	95% CI	*p*-value
IFOF mean FA	-0.10	-2.2, 2.0	0.924			
AF mean FA*	3.2	-0.049, 6.5	0.053	1.3	-1.9, 4.6	0.421
Group						
Affected	—	—		—	—	
Healthy	2.5	0.62, 4.5	**0.010**	2.3	0.20, 4.3	**0.032**
Sex						
Female	—	—				
Male	3.6	-0.26, 7.4	0.067			
Age	-0.05	-0.20, 0.11	0.557			
Diagnosis						
GBM	—	—		—	—	
LGG	-1.3	-5.8, 3.3	0.577	-1.5	-6.0, 3.1	0.526
Meningioma	22	16, 28	**< 0.001**	22	16, 28	**< 0.001**
Met	5.3	-0.04, 11	0.052	5.1	-0.15, 10	0.057
Side						
Left	—	—				
Right	0.60	-3.6, 4.8	0.778			

Significant* p*-values are highlighted in bold

**Table 3:** Univariable and multivariable mixed-effects linear regressions investigating the variables associated with IFOF volume (cm3). (*) AF mean FA betas are reported per 0.1 increase

Outcome: IFOF volume (cm3)	Univariable			Multivariable		
Characteristic	Beta	95% CI	*p*-value	Adj. Beta	95% CI	*p*-value
IFOF mean FA	1.8	-0.49, 4.0	0.125			
AF mean FA*	12.6	9.5, 15.7	**< 0.001**	9.0	6.2, 11.9	**< 0.001**
Group						
Affected	—	—		—	—	
Healthy	10	8.4, 12	**< 0.001**	8.4	6.4, 10	**< 0.001**
Sex						
Female	—	—				
Male	1.9	-1.1, 4.8	0.209			
Age	-0.16	-0.27, -0.05	**0.006**	-0.03	-0.16, 0.10	0.611
Diagnosis						
GBM	—	—		—	—	
LGG	6.1	2.3, 9.9	**0.002**	4.2	-0.26, 8.6	0.065
Meningioma	-8.2	-13, -3.3	**0.001**	-9.6	-14, -4.9	**< 0.001**
Met	-2.3	-6.7, 2.1	0.308	-2.9	-7.1, 1.3	0.172
Side						
Left	—	—				
Right	-1.5	-4.7, 1.8	0.376			

Significant *p*-values are highlighted in bold

**Table 4:** Univariable and multivariable mixed-effects linear regressions investigating the variables associated with IFOF mean length (mm). (*) AF mean FA betas are reported per 0.1 increase

Outcome: IFOF mean length (mm)	Univariable			Multivariable		
Characteristic	Beta	95% CI	*p*-value	Adj. Beta	95% CI	*p*-value
IFOF mean FA	-0.51	-2.6, 1.6	0.629			
AF mean FA*	6.8	3.5, 10.1	**< 0.001**	2.3	-0.95, 5.6	0.164
Group						
Affected	—	—		—	—	
Healthy	5.7	4.2, 7.3	**< 0.001**	5.2	3.5, 7.0	**< 0.001**
Sex						
Female	—	—				
Male	2.6	-4.5, 9.7	0.472			
Age	0.06	-0.22, 0.34	0.658			
Diagnosis						
GBM	—	—		—	—	
LGG	-1.1	-9.4, 7.3	0.802	-1.4	-9.8, 7.0	0.746
Meningioma	41	31, 52	**< 0.001**	41	30, 52	**< 0.001**
Met	9.4	-0.33, 19	0.058	9.2	-0.54, 19	0.064
Side						
Left	—	—				
Right	0.85	-6.9, 8.6	0.829			

Significant *p*-values are highlighted in bold

## Discussion

This multicenter analysis explored the relationship between fractional anisotropy (FA) and morphometric parameters of two major associative white matter tracts, the inferior fronto-occipital fasciculus (IFOF) and the arcuate fasciculus (AF), in patients with different intracranial tumor histotypes. Our findings indicate that FA is significantly associated with both AF and IFOF tract volume outcomes, whereas its association with tract length was less pronounced. Moreover, tumor histotype appeared to modify these associations, highlighting potential differences in white matter microstructural and morphometric features across tumor types.

The most consistent observation across our analyses was the positive association between mean FA and tract volume. This findings are consistent with previous studies reporting that FA reflects features related to axonal organization, density and myelination, serving as a marker of white matter integrity in both physiological and pathological contexts [15–17]. Higher FA values are generally observed in more organized and intact fiber bundles, whereas lower FA value have been associated with demyelination, axonal disruption, or increased extracellular space due to tumor infiltration or edema. The associations observed in the present study, especially within the tumor-affected hemisphere, suggest that FA can serve as a sensitive imaging biomarker of peritumoral white matter disruption.

Interestingly, while FA was significantly associated with AF and IFOF volumes, its associations with tract length was modest or nonsignificant after adjustment. This discrepancy may reflects the differences in the biological determinants of these two morphometric features. Tract volume is closely related to the number and density of reconstructed streamlines and may therefore be more strongly associated with diffusion anisotropy, whereas tract length may be influenced by geometric distortion or compression induced by mass effect. Accordingly, microstructural alterations reflected by FA may be more closely associated with volumetric changes than with tract shortening, as also suggested by previous DTI studies in glioma [18, 19].

An important methodological consideration is that FA is not entirely independent from tractography-derived morphometric measures. Since deterministic tractography uses FA thresholds during streamline propagation, lower FA values may reduce the number of reconstructed streamlines and consequently the estimated tract volume. Therefore, the observed association between FA and tract volume likely reflects both biological alterations of white matter microstructure and technical characteristics of the tractography process itself. Accordingly, these findings should be interpreted as associations rather than evidence of a direct effect of FA on tract morphology.

The interaction between FA and tumor histotype provide additional insight into potential differences in white matter involvement across tumor types. Specifically, the association between FA and IFOF volume appeared stronger in gliomas (both GBM and LGG) than in metastases or meningiomas. This finding is consistent with the known infiltrative growth pattern of gliomas and may reflect distinct mechanisms of tumor-WM interaction (progressive impact on microstructural integrity, leading to a proportional decrease in tract volume as FA declines). In contrast, metastases and meningiomas, traditionally considered *non-infiltrative*, tend to compress or displace fibers without significantly altering their diffusion anisotropy until later stages [20]. These findings reinforce the idea that white matter involvement by tumors lies on a spectrum, modulated by histological phenotype, growth kinetics, and peritumoral microenvironmental response.

Meningiomas, in particular, demonstrated distinctive morphometric behavior, being associated with greater tract length but lower volume compared with GBM. This may reflect fiber stretching along the dural interface, increasing apparent streamline elongation while reducing fiber density due to partial volume and tractography artifacts. Similar mechanical effects have been observed in previous morphometric studies of extra-axial tumors, emphasizing that deterministic tractography may not fully disentangle true microstructural injury from deformation-related signal alterations [21–23].

The side-related differences observed in both tracts, where the healthy hemisphere consistently showed higher FA, larger volumes, and longer tracts, further emphasize the local impact of the tumor and its peritumoral effects. Although part of this asymmetry may result from manual ROI placement or registration variability, the consistent directionality across tracts and histotypes strengthens its biological plausibility. Tumor-induced infiltration, vasogenic edema, and axonal compression are all known contributors to FA decrease and streamline loss in the affected hemisphere [19, 20].

The correlation analysis between delta variables (healthy minus affected side) revealed only weak to moderate relationships between FA and morphometric parameters, although significant for IFOF volume. This modest correlation suggests that FA variations only partially account for morphometric changes, implying additional factors, such as extracellular matrix remodeling, angiogenesis, or local inflammatory responses, contribute to white matter alterations. This observation aligns with recent connectomic and histopathologic models describing tumor-white matter interactions as complex, multifactorial processes beyond simple diffusion changes [3, 24].

From a clinical standpoint, these results support the potential role of integrating FA into preoperative tractographic assessment. FA alterations may provide a complementary information regarding tract integrity even when the fascicle remains anatomically continuous on tractography. This could inform surgical planning by identifying regions showing greater structural white matter alteration on diffusion imaging. However, because intraoperative mapping data and postoperative functional outcomes were not available, no direct conclusions regarding functional integrity or resilience can be drawn from the present study. Moreover, the stronger coupling between FA and morphometric measures in gliomas supports its potential role in differentiating infiltrative from *non-infiltrative* lesions, thereby refining patient-specific surgical strategies [25, 26].

Several methodological considerations should be acknowledged. Deterministic tractography, though clinically practical, can underestimate fiber complexity, particularly in regions with crossing fibers or severe FA reduction, leading to potential underrepresentation of tracts in infiltrated areas [23, 27]. Variability in MRI field strength and diffusion directions across centers may also have influenced FA estimation. Nevertheless, the large sample size and multicenter design enhance the robustness and generalizability of our findings, mirroring the heterogeneity of real-world neuro-oncological imaging. Finally, the cross-sectional nature of our analysis precludes causal inference; longitudinal DTI monitoring would be valuable to determine whether FA decline precedes or merely accompanies volumetric tract changes.

An additional limitation relates to scanner and acquisition heterogeneity. Examinations were acquired across multiple institutions using both 1.5T and 3T scanners and different diffusion sampling schemes. Because of the retrospective design and the uneven distribution of tumor histotypes among centers, scanner or center effects were not included in the statistical models. Consequently, residual variability related to acquisition differences may have influenced FA and tractography-derived measurements. Future prospective studies employing harmonized acquisition protocols and dedicated harmonization strategies will be necessary to further validate these findings.

Another important limitation concerns the lack of histological validation of tractography findings. However, direct histological confirmation of structures presumed to represent eloquent white matter tracts would require targeted sampling of tissue considered to belong to functional white matter pathways. Such an approach would not be ethically justifiable in routine clinical practice, as it could expose patients to unnecessary neurological risk without any direct therapeutic benefit. Consequently, this limitation is inherent to most in vivo tractography studies investigating white matter anatomy.

Moreover, this was conceived as a neuroradiological study focused on preoperative imaging biomarkers and white matter tract characteristics. Therefore, intraoperative mapping findings and postoperative functional outcomes were not included in the study design, and direct correlations between tractographic alterations and functional status could not be assessed.

Ultimately, our results support a paradigm shift in the interpretation of white matter involvement in brain tumors. Beyond spatial proximity, tumor-tract interaction should be conceptualized as a dynamic interplay between microstructural integrity, mechanical deformation, and histotype-specific biological behavior. In this framework, FA may represent a useful imaging parameter associated with tumor-related white matter alterations, although its role as biomarker requires further validation in prospective studies.

## Conclusion

FA was significantly associated with the volume of the AF and IFOF, whereas its association with tract length was limited. These associations appeared stronger in gliomas, indicating that infiltrative tumors more profoundly alter white matter integrity than *non-infiltrative* ones. These findings indicate that FA may provide complementary information regarding tumor-tract interactions and support its integration into preoperative assessment to better characterize white matter involvement and guide surgical planning. Further longitudinal and prospective studies are warranted to clarify its potential role as an imaging biomarker and its relevance for preoperative assessment and surgical planning.

## Data Availability

No datasets were generated or analysed during the current study.
